# Systematic review of potential developmental and reproductive toxicity of microplastics

**DOI:** 10.1093/toxsci/kfaf108

**Published:** 2025-08-05

**Authors:** Seneca Fitch, John Rogers, Sue Marty, John Norman, Steffen Schneider, Erik Rushton, Daniele Wikoff, Robert Ellis-Hutchings

**Affiliations:** ToxStrategies, Asheville, NC 28801, United States; ToxStrategies, Asheville, NC 28801, United States; Dow, Midland, MI 48674, United States; American Chemistry Council, Washington, DC 20002, United States; BASF, Ludwigshafen am Rhein, 67056, Germany; LyondellBasell Industries, Rotterdam, 3013AA, the Netherlands; ToxStrategies, Asheville, NC 28801, United States; Dow, Midland, MI 48674, United States

**Keywords:** systematic review, microplastics, developmental toxicity, reproductive toxicity, critical appraisal

## Abstract

Plastic microparticles, a form of microparticles commonly referred to as microplastics (MP), have been the focus of increasing interest for understanding potential human and ecological impacts, including the development of health-based benchmark values. This systematic review critically evaluates 24 mammalian studies reporting reproductive and developmental outcomes, a disproportionately focused research area, with a particular focus on methodological rigor and risk of bias. Fit-for-purpose aspects of selection, performance, and attrition bias were integrated into the critical appraisal to better understand the potential bias studies may have across these domains. All studies received a tier III rating based on the National Toxicology Program’s Office of Health Assessment and Translation framework, indicating a high risk of bias and insufficient reliability for risk assessment. Key issues identified across the body of evidence include poor exposure characterization, inadequate outcome assessment, lack of validated test guidelines, and failure to account for critical reproductive parameters such as estrous cycle monitoring and sperm analysis standards. Additionally, discrepancies in the particle characterization and homogeneity of the test material limit comparability and reproducibility across studies. This work highlights the current limitations in the body of evidence in terms of internal and construct validity, which preclude any conclusions on MP-related reproductive toxicity, and details a path forward for investigators to consider in future research.

Plastic microparticles, commonly referred to as microplastics (MPs), are solid polymer-containing particles with a wide range of physicochemical properties. A growing number of studies are reporting the occurrence of MPs in the environment, including geographies as remote as Antarctica (Aves et al. 2022). MPs have also been found in air, water, food, and beverages (WHO 2022). As such, there is great interest in understanding the potential human health risks of exposure to these particles and developing health-based benchmarks. However, the World Health Organization’s recent report on dietary and inhalation exposure to nano- and MP particles concluded that data on human exposure, epidemiological observations, dosimetry and biokinetics, and toxicological effects were sparse (WHO 2022).

The complexity and heterogeneous physical characteristics of these materials present a novel challenge for the traditional framework of chemical risk assessment (Coffin et al. 2022; Gouin et al. 2022; Koelmans et al. 2022). Due to this complexity, there is little scientific or regulatory consensus on the definition of MPs in terms of composition and dimensions or size (WHO 2022). Many definitions relevant to environmental and human health suggest a range of 1 µm to 5 mm (Coffin et al. 2022; WHO 2022; ISO 2023), though some such as the EU REACH Restriction on Microplastics (EU 2023) have designated MPs range to be 0.1 µm to 5 mm based on practical sampling and detection methods.

Despite these limitations in defining MPs, regulatory initiatives such as California’s legislative mandates (SB 1422 and SB 1263) have underscored the need for toxicity values to properly evaluate and manage potential health effects of MPs. These mandates have resulted in a review of the scientific literature of MPs to determine potential health effects, which to date has disproportionately focused on developmental and reproductive toxicity study designs (Hu et al. 2024) compared with general toxicity or other health effect endpoints. Stemming from this review, the California State Water Resources Control Board’s expert panel assessed approaches to developing thresholds for when biological effects are likely to be triggered as a result of MP exposure; however, data quality concerns precluded the panel’s derivation of health-based benchmark values (Coffin et al. 2022). These data quality findings were consistent with the conclusion of the WHO report that most of these studies lack sufficient quality control for the purposes of conducting a full quantitative risk assessment. For instance, although some studies suggest adverse outcomes such as lower anti-Müllerian hormone (AMH) concentrations (An et al. 2021), these findings often lack sufficient methodological rigor. Nonetheless, researchers have attempted to establish non-regulatory screening benchmarks based on point-of-departures for these reproductive measures, further emphasizing the urgent need for standardized, high-quality research (Coffin et al. 2022).

As the body of evidence has continued to grow, more information has become available regarding methodological attributes unique to the conduct of studies involving MPs (de Ruijter et al. 2020; Gouin et al. 2022). Building on the quality assurance/quality control (QA/QC) criteria developed and applied to the ecotoxicology evidence stream by de Ruijter et al. (2020), Gouin et al. (2022) formally established the “Nano- and Microplastic Particles Toxicity Screening Assessment Tool” (NMP-TSAT) for the screening and prioritization of MP studies evaluating potential human health risks. The NMP-TSAT provides guidance for the evaluation of the methodological rigor of study designs. The application of the tool by Gouin et al. (2022) highlighted significant variability in the quality of studies reporting health outcomes of MP exposure, further complicating any synthesis of evidence for regulatory use.

The objective of this systematic review (SR) is to systematically investigate the body of evidence related to MP exposure and reproductive and developmental toxicity, and in particular, the potential risk of bias (RoB) underlying the study data, by applying evidence-based toxicology methods employed by authoritative bodies (OHAT 2015a; EPA 2022). Herein, we identify and evaluate mammalian reproductive studies assessing the potential effects associated with MPs in the context of hazard and risk assessment (including dose–response) with an emphasis on the refinement of critical appraisal approaches to evaluate the internal and construct validity of each relevant study.

## Materials and methods

**Fig. 1.**
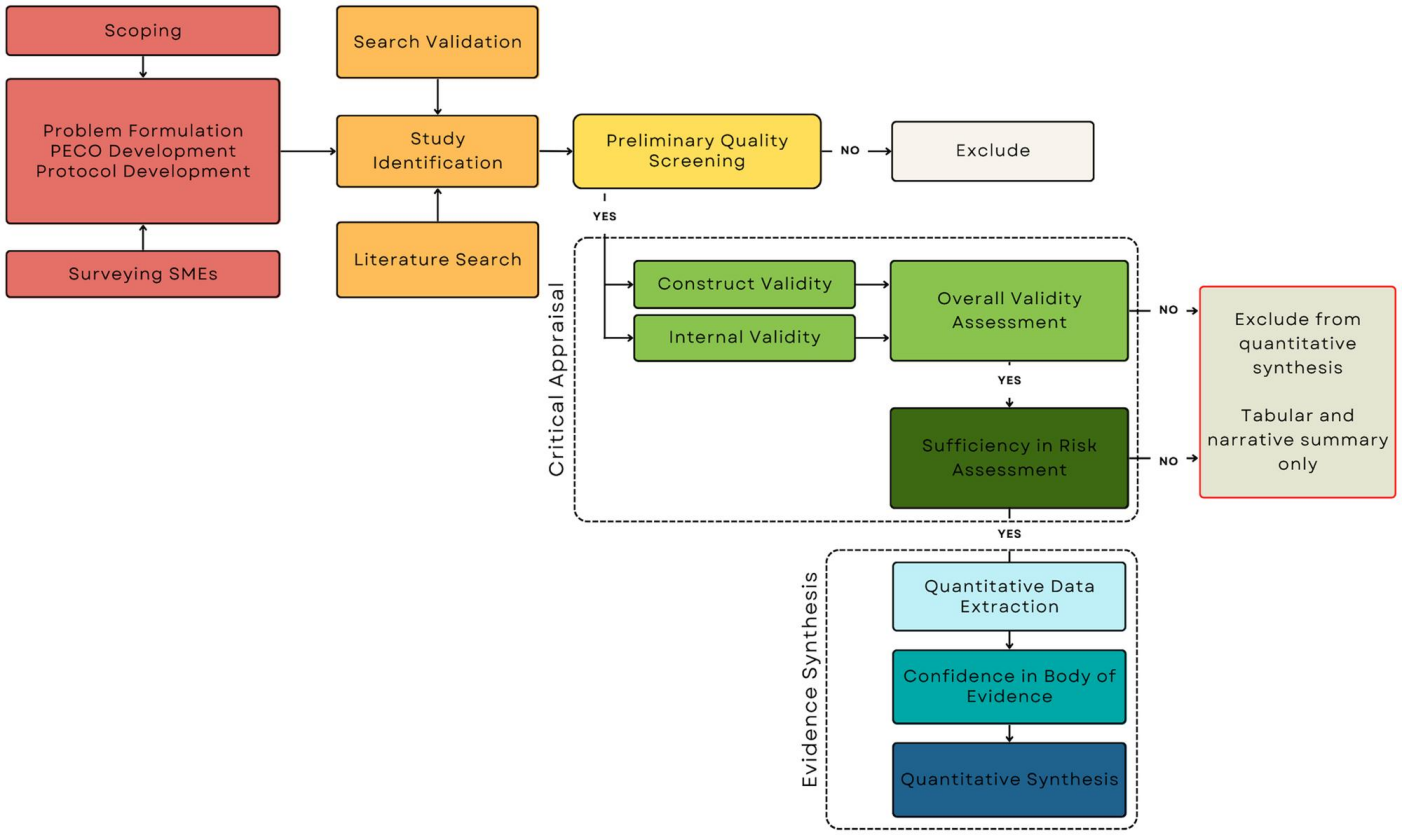
Depiction of each stage of the workflow implemented in the process of this SR.

This investigation followed a SR workflow previously reported in a protocol, prospectively registered and available to the public via the Open Science Framework (Fitch et al. 2022) (https://osf.io/27esw). The only deviation to the protocol that impacted the inclusion of studies involved refining the particle size (described below), which made the review more inclusive. The workflow is closely aligned with the National Toxicology Program’s Office of Health Assessment and Translation (NTP OHAT) Handbook for Conducting a Literature-Based Health Assessment Using OHAT Approach for Systematic Review and Evidence Integration (OHAT 2015a) as well as the World Health Organization (2021) and EPA (2022) guidance on conducing SR for risk assessment, with additional QA/QC concepts for MPs reported by de Ruijter et al. (2020) and Gouin et al. (2022). In addition to the workflow, the protocol describes the research team, consisting of the SR facilitation team and Subject Matter Experts (SMEs). The SMEs were chosen based on their specific expertise in toxicology and MPs and as such were consulted for input and approval at each stage of the SR. Figure 1 visualizes the workflow determined a priori and reported in the protocol.

### Problem formulation and PECO statement

The research question, “What is the hazard and dose-response relationship between exposure to MPs and reproductive and developmental adverse effects in mammals?” was developed to address the objective of this investigation and inform the strategy of the SR. The eligibility criteria for inclusion in the SR based on each “PECO” element (**P**opulations, **E**xposures, **C**omparators, and **O**utcomes) are presented in Table 1. For the purposes of this assessment, MPs are defined as 0.1 µm to 5 mm, which is a slight deviation from the a priori protocol, which states an included particle range of 1 µm to 5 mm. This decision to broaden the lower range was made by the SMEs to align with the more conservative definition of the European Commission (EU 2023).

**Table 1.** Eligibility criteria defined during the problem formulation stage of the SR.

PECO element	Inclusion criteria	Exclusion criteria
**P** opulation	Human: Humans of any age or sex assessed in the following study types: case–control, cohort, and cross-sectional observational studies or controlled trials Animal: non-human, mammalian experimental animals assessed in vivo	Non-mammalian models Human or non-human mammalian species assessed in vitro, ex vivo, or in silico Human or non-human mammalian species described in secondary research; reviews; editorials/opinion pieces; conference or workshop abstracts Humans described in case studies
**E** xposure	Exposures to microplastics, defined as plastic particles 0.1 µm to 5 mm, via oral, inhalation, or dermal routes of any exposure duration and frequency At least 2 treatment groups (i.e. negative control AND 2 dose groups) OR multiple particle characteristics (e.g. multiple sizes)^a^	Intentional controlled co-exposure such as mixtures of MP and another chemical (e.g. contaminants, additives) Routes of exposure other than oral, inhalation, or dermal Doses/concentrations of test material are not reported^a^ Duration and frequency of exposure to the test material are not reported^a^ Particle size of test material is not reported^a^ Polymer type of test material is not reported^a^ Study design includes one treatment group^a^
**C** omparator	Studies that include an untreated or vehicle-exposed negative control	Studies with no appropriate comparator Studies that do not report results of the negative controls Studies that do not use concurrent controls
**O** utcomes	Outcomes related to mammalian male and female reproductive and/or developmental endpoints	Outcomes unrelated to reproductive and/or developmental endpoints

a Preliminary quality screening criteria.

Critical considerations for reliable study evaluation within the context of MPs and human health were also identified during formulation, such as those considered by the NMP-TSAT tool (Gouin et al. 2022). In this review, these aspects of the study design or minimum reporting criteria were designated as preliminary quality screening criteria. Any study not meeting these requirements at full text were excluded.

### Study identification

A literature search strategy was developed based on the PECO components of interest. Potentially relevant literature was identified by traditional citation database searching of PubMed, hand searching based on previous evaluations of MPs, and surveying the SMEs (SM, JN, ER, RE-H). Publications of high relevance identified by SMEs were also used to validate the search syntax, and amendments to key terms were made to increase search sensitivity (i.e. broadness) to reach 100% of the validation set; citations for publications used for validation are provided in Table S3. A PubMed search utilizing the validated search syntax was initially performed on December 17, 2021. Due to the accelerating pace of publications related to the objective of this study, additional search updates were performed on September 19, 2022, April 4, 2023, and September 27, 2023. Specific details of the literature search strategy (including final search syntax) are available in the protocol (Fitch et al. 2022).

### Relevance and preliminary quality screening

Publications identified in the literature search were de-duplicated via EndNote X9 (Clarivate Analytics, PA, USA) and uploaded to the systematic review software DistillerSR (Evidence Partners, Ontario, CA). Relevance and preliminary quality screening consisted of both title and abstract (TiAb) and full-text review, according to the criteria stated in Table 1. TiAb screening was performed by a single human reviewer (SF, JR, DW) and confirmed by DistillerSR’s Artificial Intelligence feature. Conflicts between the human and machine learning classifications were reviewed for relevance by a second human reviewer. If a TiAb did not report a particular inclusion criterion, it was advanced to full-text review for further consideration. Following TiAb screening, relevance and preliminary quality were confirmed at the full-text level by 2 reviewers. Studies meeting all criteria at full-text review were advanced to critical appraisal.

### Critical appraisal

It was determined during problem formulation that no single critical appraisal tool that currently exists is appropriate for the evaluation of MPs studies. For example, the NMP-TSAT (Gouin et al. 2022) is a comprehensive QA/QC tool that was developed as an initial screening approach to assess MP studies in the context of human health risk assessment, but areas of potential bias such as those described by NTP OHAT (2015b) are not adequately incorporated. To address this component of the systematic evaluation, a fit-for-purpose approach was developed for the critical appraisal of study quality based on the NMP-TSAT and OHAT’s (2015b) RoB tool. Prompting questions reported by Danopoulos et al. (2022) were also considered in the refinement and application of the RoB tool. This comprehensive approach comprised the evaluation of both internal and construct validity as defined by OHAT (2015b), as well as an assessment of adequacy for use in risk assessment (Fig. 1).

The tool adopted the domain-based formatting of the OHAT RoB tool for the assessment framework and foundational RoB concepts. Next, critical fields as measured by the NMP-TSAT were mapped to the relevant OHAT RoB metric and integrated into the guidance (Table S1). Key questions for this internal validity assessment were selected based on the need for confidence in the accuracy of the exposure characterization (question 8a, test agent characterization; question 8b, characterization of test agent administration) and outcome assessment (question 9, assessment methods) domains. Other questions addressed RoB based on potential for bias in randomization and allocation of animals to study groups, appropriate and consistent experimental conditions across groups, blinding of research personnel to dose group during the study, and completeness of data without attrition or exclusion. Table 2 provides an overview of the RoB domains, questions, and type of information evaluated, respectively. These concepts are derived from the OHAT’s (2015b) RoB tool and NMP-TSAT (Gouin et al. 2022). The detailed guidance for the application of the tool by critical appraisers, including fit-for-purpose revisions, is available in Table S2.

**Table 2.** Overview of integration of OHAT’s RoB tool (OHAT 2015b) and NMP-TSAT (Gouin et al. 2022).

Domain	OHAT RoB question	Example of parameter(s) evaluated
Selection	1. Was administered dose or exposure level adequately randomized?	Randomization of animals (including method)
	2. Was allocation to study groups adequately concealed?	Blinding of exposure or treatment group allocation
Performance	5a. Were experimental conditions identical across study groups?	Concurrent controls, animal numbers in group housing, potential confounding, or modifying variables
	5b. Were experimental factors such as vehicle, feed, and housing appropriate for the experimental model?	Test medium/vehicle, satisfactory descriptions of feeding and housing of test animals (particularly related to phytoestrogen content), adequate light–dark cycles, temperature, and humidity; non-particle aspects of the test materials should also be reported (e.g. solvent/suspension aids, purity/impurities, non-plastic additives such as preservatives or fluorophore)
	6. Were the research personnel blinded to the study group during the study	Concealment or blinding to the treatment group (to the extent possible) for the duration of the experiment
Attrition	7. Were outcome data complete without attrition or exclusion from analysis?	Loss of animals, incomplete outcome data
Detection	**8a. Can we be confident in the exposure characterization? (test agent/particle characterization)**	Particle source, surface chemistry, purity, microbial contamination, particle stability, storage, concentration units; includes consideration of independent analytical verification of particle characterization
	**8b. Can we be confident in the exposure characterization? (test agent administration)**	Exposure was consistently and concurrently administered across treatment groups, administered dose/concentration, homogeneity of exposure, administration route, frequency and duration of exposure, or other confirmation of internal dose
	**9. Can we be confident in the outcome assessment?**	Method of assessment (valid and reliable), duration of exposure is appropriate for outcome evaluation, sample size, appropriate measures of stress, blinding of outcome assessors, use of negative (vehicle) controls
Selective Reporting	10. Were all measured outcomes reported?	Results of primary and secondary outcomes listed in methodology are reported with sufficient detail to perform independent analyses
Other Sources of Bias	11. Were statistical methods appropriate?	Statistical methodology is reported; units of analysis in developmental and reproductive toxicity (DART) studies should be litters for applicable outcomes

Full mapping of criteria to each RoB metric is provided in Tables S1 and S2. Bold text = key domain.

Each metric was scored by a reviewer (SF, JR, SM, SS, ER, RE-H) with 1 of 4 responses: Definitely low RoB (++), probably low RoB (+), probably high RoB or not reported (−), or definitely RoB (−−). A second reviewer reviewed the responses and associated justification of the first reviewer. If there was disagreement on the bias response, a third reviewer weighed in on the critical appraisal. Studies were then given an overall internal validity appraisal, following OHAT’s three-tier system, based on this assessment (OHAT 2015a). In this system, tier I studies are considered to have low RoB (particularly among key questions), whereas tier II studies have high RoB. Studies that do not meet the criteria for Tiers I or III are assigned to tier II. Briefly, tiers are defined as follows:

- Tier I: A study is rated as “definitely low” or “probably low” RoB for most key elements and have most other applicable items answered, “definitely low” or “probably low” RoB.
- Tier II: A study that neither meets the criteria of tier I or tier III.
- Tier III: A study is rated as “definitely high” or “probably high” RoB for most key elements and have most other applicable items answered, “definitely high” or “probably high” RoB.

Next, construct validity was assessed for each publication (JR). Here, construct validity is assessed based on the study design relative to the stated PECO. In other words, this is a measure of the extent to which the study or test accurately assesses the measures of interest. Although construct validity is recognized to be an important aspect of systematic reviews, no frameworks currently exist for appraising such within the study types included in this investigation (Wikoff et al. 2020). For the purposes of this assessment, construct validity was based on both the reliability of the study design in the context of harmonized guideline study protocols (e.g. OCSPP 870.3550 or OECD 421) and subject matter expertise. This metric was categorized as “high,” “medium,” “low,” or “unacceptable.” This categorization was wholly determined by SME knowledge of the measures required by harmonized guidelines appropriate for determining reproductive and developmental effects.

Finally, a determination on whether the reported data were sufficient for risk assessment was made for each publication. This evaluation was based on parameters of the NMP-TSAT tool, including the adequacy of the concentration range and relevance to environmental concentrations, effect thresholds or access to raw data for derivation of such, inclusion of multiple particle characteristics in the test material, and use of adequate statistical methods (i.e. NMP-TSAT criteria C1 to C6), as well as general concepts on sufficiency for risk assessment per OHAT (2015a) and EPA (2022). Studies that received a tier III internal validity score and/or a low construct validity score were automatically designated as insufficient for risk assessment for this validity category.

### Evidence synthesis

The body of evidence was summarized in tabular and narrative formats based on the results of the critical appraisal. High-level tabular data inventories in support of this stage were completed by SF and JR and reviewed by RE-H, SM, and JN, to document critical elements of the study, design such as exposure parameters and measured outcomes. The sequence of this workflow deviates from that reported in the protocol, where data extraction is performed prior to critical appraisal. This decision was made during the process of this SR piloting based on reviewer observations that studies not qualifying for quantitative evidence synthesis should not undergo extensive quantitative dose–response data extraction. Though the a priori protocol describes a quantitative synthesis (e.g. dose–response assessment, meta-analyses) if the data warrant, such was not achieved due to the low overall validity and sufficiency for use in risk assessment (OHAT 2015a; EPA 2022), as well as heterogeneity in study design precluding use of meta-analytical technique. As such, no quantitative data extraction was performed, and evidence was excluded from quantitative synthesis and described qualitatively in narrative summaries of study designs, outcomes, and RoB findings only.

## Results

### Study identification

**Fig. 2.**
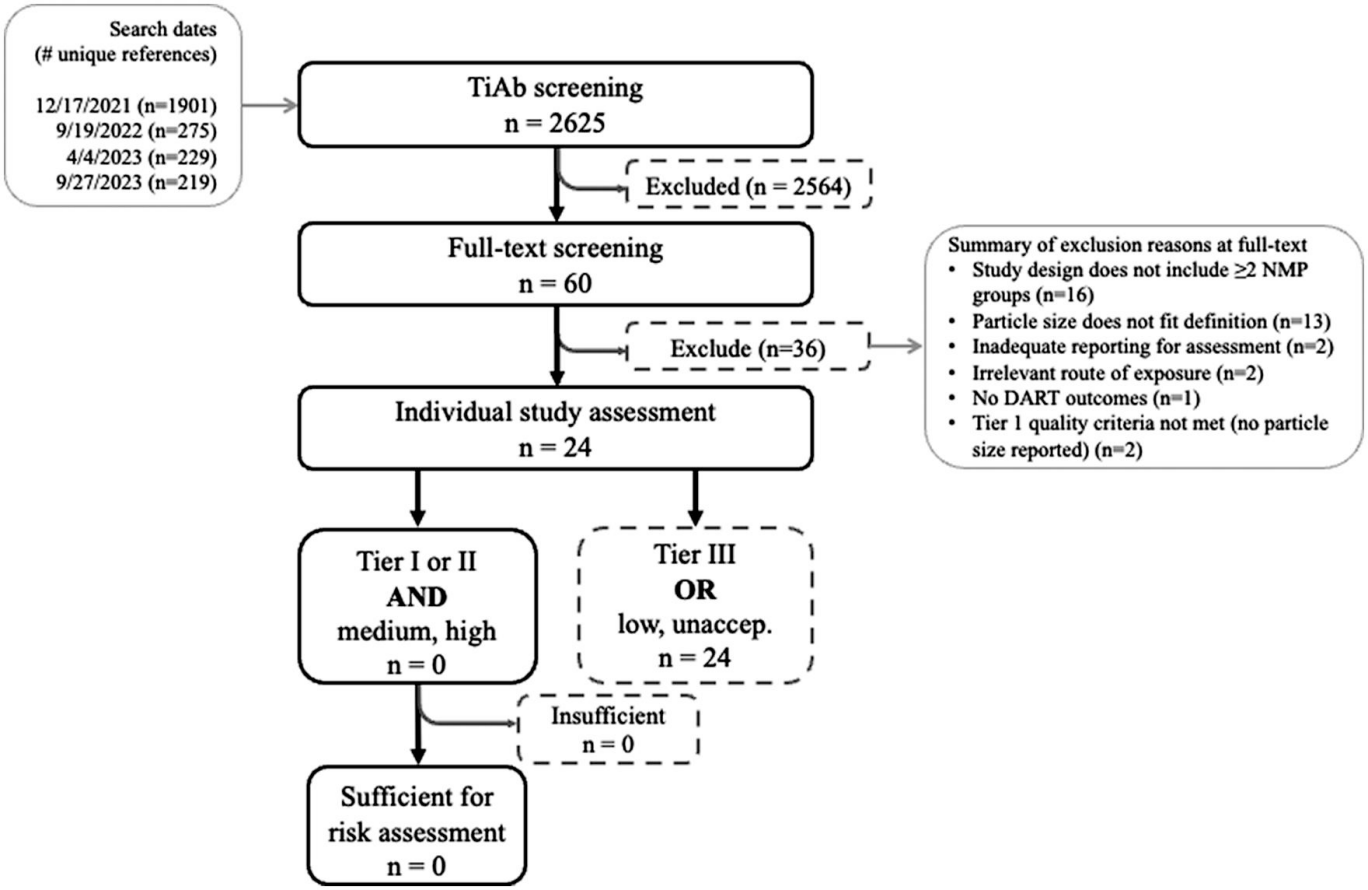
Flow chart of systematic literature review and reasons for exclusion at full-text screening.

In total, 2,625 citations were identified for review (Fig. 2). Following TiAb screening, 60 studies remained for full-text relevance and preliminary quality screening. Of these, 36 were excluded at full-text review, and 24 advanced to critical appraisal and underwent individual study assessment. The majority of studies excluded at full text were due to study designs that had a single exposure group (*n* = 16). All reviewed citations and screening decisions are available in Table S3.

### Critical appraisal and summary review of validity

**Fig. 3.**
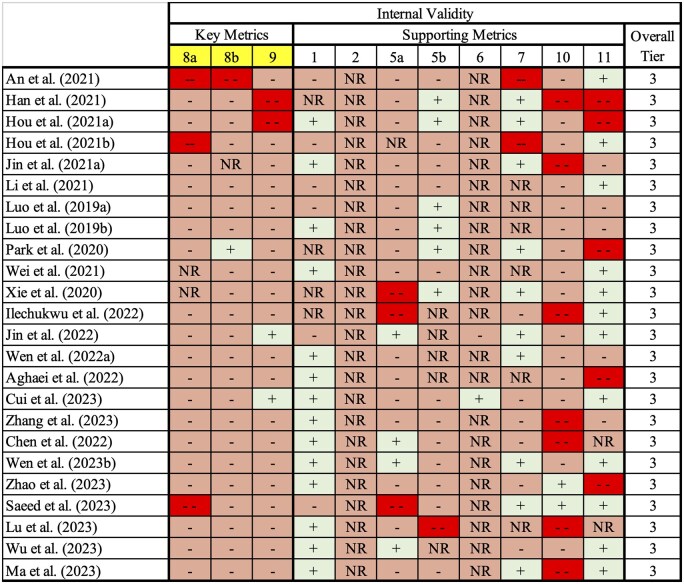
Heatmap of the results of the risk of bias assessment (light green + = probably low; light red − = probably high; dark red −− = definitely high; NR = not reported).

Twenty-four publications reporting MP (≥0.1 µm to 5 mm) exposure and developmental or reproductive outcomes were advanced to critical appraisal following full-text review. All 24 studies received an overall tier III rating (Table S4); results were aggregated into a heat map for visualization (Fig. 3). Generally, these ratings were driven by probable RoB across key domains, including lack of adequate particle characterization, low confidence in exposure characterization, and inadequate outcome assessment methods based on the discussed critical appraisal protocol.

Metrics related to blinding were largely rated as Not Reported (“NR”). Similarly, all studies were scored with “NR” for concealment of allocation to study groups (Q2). There was also a lack of reporting regarding the blinding of research personnel for the duration of the study and outcome assessments (Q6) for all but 2 studies (Jin et al. 2021; Cui et al. 2023).

None of the identified studies reported gold-standard analytical characterization of the source material or the test material (USEPA 1989; Boverhof and David 2010; OECD 2012, 2018a; Potthoff et al. 2017; ISO 2020; Gouin et al. 2022; WHO 2022; Gouin et al. 2024). Although characterization of test particles, including morphology, composition, surface features, and stability, is critical to interpreting study outcomes, significant gaps were observed in the reporting of these parameters. As such, no studies achieved “probably low” or “definitely low” RoB scores for Q8a, one of the key questions for internal validity.

Nearly all of the studies evaluated polystyrene particles (*n* = 21), with the exception of 3 studies that used polyethylene MPs (Park et al. 2020; HAn et al. 2021; Zhang et al. 2023). Of the 21 polystyrene MP studies, only 3 studies evaluated particles that were not pristine polystyrene MP spheres; this included irregular, UV-aged particles (Cui et al. 2023), manually crushed polystyrene from disposable plates (Ilechukwu et al. 2022), and cryogenic milling of polystyrene pellets (Saeed et al. 2023). Fourteen of the 21 studies (67%) administered polystyrene MPs purchased from the same supplier (Tianjin Baseline ChromTech Research Centre in Tianjin, China). The percentage of studies reporting male reproductive effects using particles from this single supplier is higher relative to those using a different supplier; these studies account for 85% of studies reporting significant effects for male reproductive endpoints. Regarding particle size, measures ranged from the minimum included (100 nm) up to 2 mm, though most of the studies tested particles ranging from 0.5 to 10µm (*n* = 19).

Critical deficiencies and limitations were also identified for key metric 8b, which interrogates confidence in exposure characterization as it relates to the administration of the test article and the reported dose. NTP OHAT RoB guidance for Q8 states, “exposure characterization should also include verification of the compound over the course of the test period”—however, none of the reviewed studies analytically verified the prepared MP test material concentrations at a single time point or over the course of the study. Other uncertainties in the exposure characterization were due to missing data, such as water intake per animal or the number of animals per cage, both of which are critical for having confidence in the exposure assessment.

Some limitations are route-specific, such as those in the 12 studies that administered particles to rodents via drinking water. As an example, consider that a 5 µm polystyrene sphere reported to have a 1.05 g × cm^−3^ density (Sigma-Aldrich) would generally be expected to sediment in a drinking water mixture (Hinderliter et al. 2010), whereas low-density particles may be buoyant in such media (Watson et al. 2016). Either situation would result in significant overall uncertainty and potentially large variations in the actual dose received by the animals. The reality is more complex as particle diffusion in the aqueous media (e.g. drinking water) will be dependent on the effective density, influenced by particle agglomeration, the ionization state of the media, and if/how the particle test material has been stabilized (Hinderliter et al. 2010). These dynamics of particle diffusion further reinforce the critical necessity of characterization in determining accurate and intended dosimetry. Though critical, consistency and homogeneity of dosing solutions were not monitored. The lack of reported water consumption data, which is required by OECD TGs for this route of exposure (e.g. OECD 2018b), presents another significant challenge in accurately determining the actual dose of MPs received by test animals. In 8 studies where MPs were administered via drinking water, the exposure concentration relied on reported nominal water concentrations rather than measured intake (Luo et al. 2019a, 2019b; An et al. 2021; Hou et al. 2021a; Li et al. 2021; Aghaei et al. 2022; Chen et al. 2023; Zhao et al. 2023). Ultimately, these unknown variables impact the reliability of dose–response assessments and introduce an unacceptable level of uncertainty. This variability in MP delivery may obscure subtle dose–response relationships, leading to either false negatives or false positives in observed outcomes. As a result, the overall confidence in the dose or exposure of animals to MPs is reduced.

Most drinking water studies reported treating MP solutions with ultrasound to ensure suspension prior to administration. However, it was frequently unclear whether ultrasound was performed on the stock solution or the prepared dose solution. Additionally, the frequency of preparation (e.g. daily, weekly) was not consistently reported, which raises concerns about particle stability and settling or aggregation over time. In a subset of these studies, authors state that water bottles were regularly shaken to help with particle dispersion over the course of the experiment. None of these studies reported analytical verification to confirm the presence and distribution of polystyrene MPs in prepared solutions during the exposure period. Additionally, shaking water bottles is likely to introduce additional periods of interference with test animals. It is unclear whether water bottles were also shaken for control groups to ensure equivalent environmental stress to all animals, which also impacts performance bias (Q5a and Q5b).

Other parameters that may have impacted the consistency and appropriateness of non-treatment-related experimental conditions (i.e. Q5a and Q5b) were often assumed, as recommended in the NTP OHAT guidance for performance bias. For example, most studies described the control group receiving “deionized water” or “normal water,” which was assumed to be the same as the vehicle for test solutions. However, several studies did not confirm whether the suspension vehicles for MPs (e.g., ethanol, ultrapure water) were comparable to the water provided to control animals. Similarly, most studies lacked reporting on whether control animals were sham-treated to account for procedural handling differences (such as shaking drinking water bottles or gavage dosing).

Other risks to study validity, such as outcome assessment quality, selective reporting, and appropriateness of statistical methods, are reported below where relevant for each outcome of interest (i.e. male reproductive effects, female reproductive effects, or developmental effects). It is important to reiterate that all studies received “Tier III” scores in critical appraisal, indicating that results should be interpreted with caution due to potential quality and reliability deficits. Although results are summarized herein for completeness, the overall reliability of these results is insufficient for risk assessment purposes.

### Male reproduction

Fourteen studies (shown in Table 3) evaluated male reproductive endpoints, such as histopathology of reproductive organs, sperm parameters, and serum hormone concentrations (Park et al. 2020; Xie et al. 2020; Jin et al. 2021, 2022; Li et al. 2021; Wei et al. 2021; Hou et al. 2021a; Ilechukwu et al. 2022; Wen et al. 2022; Cui et al. 2023; Lu et al. 2023; Ma et al. 2023; Wen et al. 2023; Wu et al. 2023). None of the studies adhered to validated protocols such as OECD TG 421, 422, or 443, which are specifically designed to provide robust and reliable assessments of reproductive toxicity; these studies ultimately received tier III reliability ratings. This limits the ability to compare findings across studies and apply them to regulatory contexts. Further, as described in the documentation for OECD (2018b) TG 443, the rat is the preferred species for male reproductive toxicity assessments. However, only 2 studies evaluated effects in rats (Li et al. 2021; Ilechukwu et al. 2022), whereas the other 11 evaluated mice. Notably, Park et al. (2020) was the only mouse study that evaluated male fertility, though data reporting for this parameter were limited. There were no significant effects on pregnancy rates or number of live births, but this assessment of reproductive function was limited (e.g. only 5 mice/sex/dose were evaluated).

**Table 3.** Study design data of included studies reporting male reproductive endpoints.

Study	Species (strain); No. animals/group	Route	Dose	Polymer	Particle size (shape)	Particle surface features	Duration	Particle Source	Endpoint
Cui et al. 2023	Mouse (ICR); *n* = 7	Unclear^a^	0, 0.01, and 1 mg/day	PS	4 to 6 µm (pristine spheres and irregular UV aged)	No information	1 week	Tianjin Baseline ChromTech Research Centre (Tianjin, China)	Testis histopathology
Hou et al. 2021a	Mouse (ICR); *n* = 10	Drinking water	0, 100, 1,000, and 10,000 µg/l	PS	5 µm (sphere)	No information	35 days	Tianjin Bestra Chromatography Technology Development Center	Organ weight (epididymis, testis) Sperm viability Sperm abnormality rate Testis histopathology
Ilechukwu et al. 2022	Rat (Wistar); *n* = 4 or 6	Diet (feed)	0, 1, 5, and 10%	PS	<2 mm (irregular)	No information	90 days	Crushed polystyrene plates purchased from a local store in Nigeria	Sperm count Sperm motility Sperm abnormality rate Sperm viability Serum testosterone Testis histology
Jin et al. 2021	Mouse (BALB/C); n = 3 or 12	Oral gavage	0 and 100 μg/l	FPS	0.5, 4, and 10 μm (sphere)	All negatively charged (Zeta potentials > −20 mV)	28 days	Tianjin Baseline ChromTech Research Centre (Tianjin, China)	Testis weight Testis histopathology Serum testosterone Sperm viability Sperm abnormality rate
Jin et al. 2022	Mouse (BALB/C); *n* = 15	Drinking water	0, 100 μg/l, and 1,000 μg/l	FPS	0.5, 4, and 10 μm (sphere)	No information	180 days	Tianjin Baseline ChromTech Research Centre (Tianjin, China)	Organ weight (epididymis, testis) Sperm viability Sperm abnormality rate Testis histology Serum hormone concentrations (testosterone, LH, FSH)
Li et al. 2021	Rat (Wistar); *n* = 8	Drinking water	0, 0.015, 0.15, and 1.5 mg/day	PS	0.5 µm (sphere)	No information	90 days	Tianjin Baseline ChromTech Research Centre (Tianjin, China)	Sperm count Sperm motility Sperm abnormality rate Testis histology
Lu et al. 2023	Mouse (C57BL/6); *n* = 6	Oral gavage	0, 10, and 40 mg/kg bw/day	PS	5 µm (sphere, assumed)	No information	60 days	Tianjin Baseline ChromTech Research Centre (Tianjin, China)	Organ weight (epididymis, testis) Testis histology Sperm count Sperm motility
Ma et al. 2023	Mouse (BALB/c); *n* = 10	Oral gavage	0, 5, and 50 mg/kg bw/day	PS and FPS	100 nm (sphere)	No information	30 days	Tianjin Baseline ChromTech Research Centre (Tianjin, China)	Organ weight (epididymis, testis) Serum testosterone Sperm counts Sperm abnormality rate Testis histopathology
Park et al. 2020	Mouse (ICR); *n* = 5 or 10	Oral gavage	0, 0.125, 0.5, 2.0 mg/d	UHMWPE	16.9 ± 1.9 µm (irregular)	Modified to contain acid and hydroxyl groups	90 days (6 days/week)	Sigma-Aldrich (Darmstadt, Germany)	Histopathology of male reproductive organs (testes, seminal vesicle)
Wei et al. 2021	Mouse (BALB/c); *n* = 10	Oral gavage	0, 20, and 40 mg/kg bw/day	PS	4 μm and 10 μm (sphere, assumed)	No information	28 days	Tianjin Baseline ChromTech Research Centre (Tianjin, China)	Organ weight (epididymis, testis) Testis histology Sperm counts Sperm abnormality rate
Wen et al. 2023	Mouse (Kunming); *n* = 10	Oral gavage	0, 0.01, 0.1, and 1.0 mg/day	PS and FPS	10 μm (sphere)	Negatively charged (Zeta potential = −17.9 mV)	35 days	Base Line ChromTech Research Centre (Tianjin, China)	Organ weight (epididymis, testis) Sperm count Sperm motility Sperm abnormality rate Testis histopathology Serum testosterone concentration
Wen et al. 2022	Mouse (C57BL/6); *n* = 7	Drinking water	0, 100, and 1,000 μg/l	PS and FPS	5 μm (sphere)	No information	90 days	Baseline ChromTech Research Centre (Tianjin, China)	Organ weight (epididymis, testis) Sperm count Sperm motility Sperm abnormality rate Testis histopathology Serum hormone concentration (FSH, LH, testosterone)
Wu et al. 2023	Mouse (C57); *n* = 5	Drinking water	0, 1, and 5 mg/kg bw/days	PS and FPS	1 µm (sphere)	No information	4 weeks	Tianjin Bestra Chromatography Technology Development Center	Testis histology
Xie et al. 2020	Mouse (BALB/c); *n* = 10	Oral gavage	0, 0.01, 0.1, and 1 mg/d	PS	5.0 to 5.9 µm (sphere, assumed)	No information	42 days	Shanghai Macklin Biochemical Co., Ltd.	Testis histopathology Sperm count Sperm abnormality rate Serum testosterone concentration

PS, unlabeled polystyrene; FPS, fluorescent polystyrene; UHMWPE, ultra high-molecular-weight polyethylene.

a Cui et al. (2023) reports 2 different routes of administration to the animals. In the abstract, authors state exposure is via intratracheal instillation. In the methods, authors state exposure is via oral gavage.

All 14 studies reported abnormal findings for histological or histopathological evaluations of the testis. Some studies failed to report methodology for the histopathological evaluations entirely (Ma et al. 2023; Wu et al. 2023). Other studies reported effects qualitatively, providing representative images of reported effects, rather than incidence or severity scores that would allow for an independent quantitative analysis as described by OECD TG 421, 422, 443 (Xie et al. 2020; Hou et al. 2021a; Jin et al. 2021, 2022; Li et al. 2021; Wei et al. 2021; Ilechukwu et al. 2022; Cui et al. 2023; Lu et al. 2023). Effects reported for other gross examinations, such as organ weight, were inconsistent, with several studies reporting no effect on relative testis (*n* = 5) or epididymis (*n* = 5) weights, whereas others reported an increase for each (*n* = 3 and *n* = 2, respectively). Rarely were absolute organ and body weights reported alongside these data, precluding an independent assessment of potential reasons for these inconsistencies. Absolute testis weight is generally preferred because testis weight is less influenced by body weight than most other tissues (Lanning et al. 2002).

Regarding sperm analyses, most studies reported effects including reduced sperm counts (*n* = 8), increased rate of sperm abnormalities (*n* = 9), and reduced motility (*n* = 4). In most instances, studies failed to report the number of sperm evaluated (total or per animal), and it is unclear if they met test guideline standards of at least 200 per sample for sperm morphology assessments (as is required in EPA test guidelines, for example USEPA 1998b). This minimum number of sperm is considered sufficient for achieving a statistically reliable estimate of sperm morphology. Smaller sample sizes could increase the likelihood of sampling bias, which could skew results and reduce confidence in conclusions. Another critical issue identified across studies reporting sperm parameters is the extraction of sperm from the whole epididymis rather than the cauda (Hou et al. 2021a; Li et al. 2021; Wen et al. 2022, 2023; Lu et al. 2023). This methodology is likely to introduce variability in counts and abnormality rates as the sperm from areas other than the cauda are still undergoing the maturation process. Other studies fail to report how sperm were collected for analysis entirely (Xie et al. 2020; Wei et al. 2021; Ilechukwu et al. 2022).

Other limitations were also noted during independent study assessments (Table S4). For example, Ilechukwu et al. (2022) report significant decreases in body weight in the mid- and high-dose groups, acknowledging potential issues due to a heavy gastrointestinal burden and difficulty in excreting MPs and decreased food absorption. Ilechukwu et al. (2022) did not report food consumption; thus, it was not possible to further evaluate the toxicological significance of the decreased body weights (and other reproduction-specific endpoints).

### Female reproduction and prenatal development

Seven studies (shown in Table 4) evaluated female reproductive endpoints (Park et al. 2020; An et al. 2021; Hou et al. 2021b; Aghaei et al. 2022; Chen et al. 2023; Saeed et al. 2023; Zhang et al. 2023). Endpoints reported in these studies included various fetal measures (e.g. fetal weight, survival, sex ratio), and maternal measures primarily focused on hormone concentrations and ovarian pathology. Many of the studies also evaluated mechanistic endpoints such as oxidative stress and inflammation biomarkers. None of the studies were performed according to validated test guidelines developed to assess female reproductive toxicity (e.g., OECD TG 421, 422, 416, 443, and 414). Serious limitations were identified across several domains, including those interrogating confidence in the outcome assessment (Q9) and selective reporting (Q10). It should be noted that none of the studies reported estrous cycle monitoring, which is critical to any female reproductive evaluation. Test animals should be confirmed as cycling regularly prior to initiation of treatment to ensure comparability of reproductive function among treatment groups (Goldman et al. 2007). Further, knowledge of the phase of the estrous cycle for the duration of the study and at necropsy is essential for correlation with histopathology in reproductive organs (OECD 2018b). Without estrous cycle data, confidence in the reliability of outcomes informing ovarian function (e.g. follicle counts) and endocrine parameters (e.g. serum hormone concentrations) is low.

**Table 4.** Study design data of included studies reporting female reproductive and prenatal developmental endpoints.

Study	Species (strain); No. animals/group	Route	Dose	Polymer	Particle size (shape)	Particle surface features	Duration	Particle Source	Endpoints of relevance
Aghaei et al. (2022)	Mouse (CD-1); *n* = 7 to 11	Drinking water	0, 0.1, 10, and 1,000 µg/L	PS	5 µm (sphere, assumed)	No information	GD0-GD17 (assumed)	Microspheres-Nanospheres (New York)	Placental organ weight Fetal weight
An et al. (2021)	Rat (Wistar); *n* = 8	Drinking water	0, 0.015, 0.15, and 1.5 mg/kg/d	PS	0.5 μm (sphere)	No information	90 days	Base Line ChromTech Research Centre (Tianjin, China)	Ovarian morphology Anti-Müllerian hormone concentration Number of growing follicles
Chen et al. (2023)	Mouse (unclear)^a^; *n* = 5	Drinking water	0, 1, and 10 mg/l	PS	100 nm (sphere)	No information	GD0-GD17	Huge Biotechnology (Shanghai)	Placental organ weight Fetal weight
Hou et al. (2021b)	Rat (Wistar); *n* = 8	Drinking water	0, 0.015, 0.15, and 1.5 mg/kg/d	PS	0.5 µm (sphere)	No information	90 days	Base Line ChromTech Research Centre (Tianjin, China)	Ovarian morphology Anti-Mullerian hormone concentration Number of growing follicles
Park et al. (2020)	Mouse (ICR); *n* = 5 or 10	Oral gavage	0, 0.125, 0.5, 2.0 mg/d	UHMWPE	16.9 ± 1.9 µm (irregular)	Modified to contain acid and hydroxyl groups	90 days (6d/week)	Sigma-Aldrich (Darmstadt, Germany)	Altered fallopian tube pathology (dams) Ovarian pathology Mating success Gestation length Pup death (PND 1) Live birth rate Body weight of pups (6 h post-birth) Sex ratio
Saeed et al. (2023)	Rat (Wistar); *n* = 6	Oral gavage	0, 2.5, 5, and 10 mg/kg/d	PS	≤1µm, average 876 nm (shape not reported)	No information	45 days	MS-Polymer (Pakistan)	Ovary weight and histopathology Hormone levels (FSH, LH, testosterone, progesterone, testosterone)
Zhang et al. (2023)	Mouse (Kunming); not reported except fertility experiments where *n* = 5	Oral gavage (assumed)	0, 0.4, 4, and 40 mg/kg/d	PE	10 to 150 μm (shape not reported)	No information	30 days	Cospheric Company	Oocyte quality Fertilization rates Litter size Hormone levels (E2, progesterone, FSH, LH)

PS, unlabeled polystyrene; FPS, fluorescent polystyrene; UHMWPE, ultra high-molecular-weight polyethylene.

a Chen et al. (2023) states, “A total of 40 seven-week-old C57BL/6 female mice (ICR) and 20 male mice were purchased from Cavens Biogle (Changzhou).” C57BL/6 and ICR mouse strains are distinct from one another and reporting in the manuscript precludes determining which strain was used during the study.

Four of the 7 studies (An et al. 2021; Hou et al. 2021b; Chen et al. 2023; Zhang et al. 2023) did not report maternal/dam body weight, an outcome measure required by OECD TGs to aid in the interpretation of treatment-related effects in offspring. In 2 of these, authors report measuring body weight but do not report results. The omission of these data limits the ability to evaluate any potential systemic toxicity and potential impact on reported effects such as decreases in fetal or pup body weights (Chen et al. 2023; Zhang et al. 2023). Similarly, 2 studies measuring ovarian outcomes failed to measure body weights or ovary weights (An et al. 2021; Hou et al. 2021b). Although studies reported effects on the number of growing follicles and serum AMH concentrations, the lack of accompanying organ weights does not allow confident toxicological interpretation of these observations.

Other serious issues were identified with An et al. (2021) and Hou et al. (2021b). During the critical appraisal of these studies, it was determined that Fig. 1A to C in both publications are the same. Authors were contacted for additional information and generously provided it to S. Fitch. Based on communications with the corresponding author (personal email communications, February 2023), 64 total animals from both An et al. (2021) and Hou et al. (2021b) were “used at the same time.” It is unclear how these animals were selected for treatment and division between the 2 research studies. As such, for the purposes of reporting, these would be most appropriately considered as a single study rather than 2 independent studies. In a review of the raw data collected for AMH concentrations, sample numbers reported in publications did not match those as shown in the raw data. For example, An et al. (2021) report that AMH concentrations were measured in “eight [animals] from each group”; however, the raw data only contains concentrations for 7 animals. Similarly, Hou et al. (2021b) report “data from six samples” but appear to calculate the mean from 8 animals. Due to these issues, these studies were determined to be unreliable for risk assessment and assigned an overall tier III category.

### Postnatal development

Developmental effects in offspring following in utero exposure were evaluated by 6 studies (Table 5) (Luo et al. 2019a, 2019b; Park et al. 2020; HAn et al. 2021; Zhang et al. 2023; Zhao et al. 2023). None of the studies were performed according to validated test guidelines developed, at least in part, to assess developmental toxicity (e.g. OECD TG 414, 421, 422, 426, and 443). A critical study design element of developmental studies is the use of the litter as the statistical unit of analysis for offspring (i.e. where “n” is the number of litters, not the number of fetuses or neonates assessed). This is necessary for appropriate statistical comparisons among the experimental groups; using the number of fetuses or neonates as the statistical unit inflates the sample size and increases the risk of false positives. This resulted in a “Definitely High” RoB rating for the Other Sources of Bias domain (Q11) for 3 studies that failed to use the litter as the statistical unit (Park et al. 2020; HAn et al. 2021; Zhao et al. 2023). Three other studies received “Probably High” RoB ratings for lack of evidence that the litter was the unit of statistical analysis (Luo et al. 2019a, 2019b; Zhang et al. 2023). This important statistical flaw reduces the confidence in the body of literature reporting developmental measures.

**Table 5.** Study design data of included studies reporting postnatal developmental endpoints.

Study	Species (strain); No. animals/group	Route	Dose	Polymer	Particle size (shape)	Particle surface features	Duration	Particle Source	Endpoint
HAn et al. (2021)	Mouse (strain not reported); *n* = 3	Intratracheal instillation	0, 6, and 60 µg/d	PE	10 to 45 µm (sphere)	No information	GD10-PND7 (approximately every other day)	Cospheric (Santa Barbara, CA, USA)	F1 organ weight and histopathology
Luo et al. (2019a)	Mouse (ICR); *n* = 3 to 6	Drinking water	0, 100, and 1,000 µg/l	PS	5 µm (sphere, assumed)	No information	Dams, GD0(assumed) - PND21 F1/F2 exposed through PND 42 or 280	Microspheres-Nanospheres (New York)	Fetal growth (F1, F2) F1 liver weights F1 serum indexes (glu, pyr, tch, tg, ldl-c, hdl-c, nefa)
Luo et al. (2019b)	Mouse (ICR); *n* = 5 to 6	Drinking water	0, 100, and 1,000 µg/l	PS	0.5 μm and 5 μm (sphere, assumed)	No information	GD0- parturition (postnatal day 0)	Microspheres-Nanospheres (New York)	F1 survival F1 sex ratio F1 BW at PND 42 F1 relative liver weight at PND42
Park et al. (2020)	Mouse (ICR); *n* = 5 or 10	Oral gavage	0, 0.125, 0.5, 2.0 mg/d	UHMWPE	16.9 ± 1.9 µm (irregular)	Modified to contain acid and hydroxyl groups	90 days (6 days/week)	Sigma-Aldrich (Darmstadt, Germany)	F1 pup body weight F1 pup immunological effects
Zhang et al. (2023)	Mouse (Kunming); not reported except fertility experiments where *n* = 5	Oral gavage (assumed)	0, 0.4, 4, and 40 mg/kg bw/day	PE	10 to 150 μm (sphere, assumed)	No information	GD0—PND21	Cospheric Company	F1 birth weight F1 survival F1 ovarian weight F1 female fertility (in vitro fertilization rates)
Zhao et al. (2023)	Mouse (ICR); *n* = 5 litters at PND35, *n* = 5 litters at PND70	Drinking water	0, 0.5 mg/l, 5 mg/l, and 50 mg/l	PS	0.5 µm (sphere)	No information	GD1—PND35 GD1—PND70	Base Line ChromTech Research Centre (Tianjin, China)	F1 balanopreputial separation F1 body weight F1 testis weight/organ coefficient F1 anogenital distance/index F1 hormone concentrations (testosterone, inhibin B, FSH, LH F1 testis histopathology F1 male fertility (mating success, sperm parameters)

PS, unlabeled polystyrene; FPS, fluorescent polystyrene; UHMWPE, ultra-high-molecular-weight polyethylene.

It is possible that the lack of consideration for litter of origin provides an explanation for the lack of consistency in reported results, such as F1 body weight. Across the body of evidence, authors report no effect on F1 body weight (Luo et al. 2019a, 2019b), increases at the mid- or high-dose (HAn et al. 2021; Zhao et al. 2023), or a decrease at the high dose (Zhang et al. 2023). Park et al. (2020) report significant pair-wise effects but no clear trend, with a significant increase from control at the low dose and a significant decrease from control at the high dose.

Methods in these studies were generally limited or directly contradicted results, creating further uncertainty. For example, Park et al. (2020) state that groups contained 15 females/dose, but footnotes of figures state *n* = 10 for maternal parameters and *n* = 5/dose for mating experiments. Across studies, insufficient sample sizes often compromised clear interpretation of study data, further increasing uncertainty. Additionally, the majority of these studies also lacked reporting of important endpoints needed to interpret the study outcomes, including maternal body weight (Luo et al. 2019a, 2019b; Zhang et al. 2023; Zhao et al. 2023) and fetus or pup viability (Luo et al. 2019a; HAn et al. 2021; Zhao et al. 2023).

### Evidence synthesis summary

Due to significant methodological limitations, including inconsistent exposure characterization, poor outcome assessment, and lack of adherence to validated guidelines (e.g. OECD TGs), none of the 24 studies advanced to the evaluation of sufficiency for risk assessment. Key bias metrics (Q8a, Q8b, Q9) consistently received ratings of “probably high” or “definitely high,” reflecting critical concerns about internal validity. Specific issues included inconsistent reporting of body weight and estrous cycle data, inappropriate sperm collection methods, insufficient sample sizes, and failure to use litter as the statistical unit in developmental and reproductive studies. NTP OHAT defines high RoB as a scenario where there is a significant potential for bias in study design, conduct, or reporting that compromises the validity or reliability of the findings. This includes when methodological issues introduce systematic errors that cannot be easily corrected or accounted for in the analysis.

As such, all of the studies identified herein were assigned a tier III based on OHAT’s evaluation process, and their findings are likely to be unreliable in terms of understanding the true effect of an exposure. In a regulatory context, these would translate to a Klimisch Score of 3 (“Not Reliable”) or 4 (“Not Assignable”) (Klimisch et al. 1997). In terms of regulatory acceptance, these are often excluded or given minimal weight in regulatory decision-making. Characterizing the reproductive and developmental toxicity of MPs based on this body of evidence is not advised due to significant variability in study designs, test materials, and methodological rigor. Although potential developmental and/or reproductive effects cannot be ruled out, the current body of evidence lacks the reliability and consistency needed for a confident assessment of hazard or quantitative evaluation of potential risks.

## Discussion

This systematic review evaluated 24 studies investigating the potential reproductive and developmental toxicity of MPs in mammals. The critical appraisal revealed significant flaws in study design and reporting, with a high RoB across key areas, making it difficult to use the results for risk assessment purposes. Many of the issues, such as inconsistent exposure characterization, lack of verification of dosing solutions, and missing data on crucial endpoints, such as estrous cycle monitoring and litter-based analyses, directly affect the ability to assess dose–response relationships with confidence. As a result, the current evidence is not reliable enough to identify causative hazards, establish meaningful toxicological thresholds, or guide regulatory decision-making.

A recent rapid systematic review by Chartres et al. (2024) examined many of the same studies as evaluated here and reported similar biases in the literature. Chartres et al. (2024) used the Navigation Guide systematic review methodology that differs from the current analysis based on the OHAT methodology in 2 critical areas. First, OHAT includes the detection bias domain that examines critical questions about exposure characterization. Consistent exposure to any small particle is difficult to achieve, and MPs have additional concerns regarding size, shape, polymer type, additive, manufacturing contamination or processes, surface charge, etc., which may affect results and the ability to extrapolate the results to other studies (Gouin et al. 2022; Koelmans et al. 2022; Boettcher et al. 2023; Shen et al. 2023). Including this parameter in their critical evaluation would likely change the RoB score for many, if not most, of the studies examined by Chartres et al. (2024). Second, the Navigation Guide method assumes experimental animal data are of “high” quality because they are comparable to human randomized controlled trials (RCTs), and GRADE designates RCTs with a high rating before any modifications due to bias (Guyatt et al. 2011; Woodruff and Sutton 2014). RCTs are considered high-quality studies in part because of the methodological standards scientists must adhere to. It is inconceivable that an Institutional Review Board would allow the administration of an intervention that had not been characterized and the dosing parameters confirmed. Beginning the assessment with no presumed rating in this case, as the OHAT process does, or carefully considering whether exposures were properly characterized, would have likely changed the rating of the quality of evidence integrated to arrive at the conclusion. Regardless, both systematic review paradigms provide valuable information to researchers by identifying areas on which to focus their efforts on and suggesting ways to improve to the quality of the literature.

Several limitations in the body of evidence for use in human health risk assessment are called to attention when comparing the test materials utilized to those identified in drinking water sources as reviewed by Danopoulos et al. (2020). For example, most studies evaluated herein tested the effects of pristine monodispersed PS microspheres in experimental animal test systems. Although humans may be exposed to non-pristine microparticles of this polymer type, evaluations of drinking water (a common pathway for exposure) suggest ingestion of other polymer types, such as polyethylene terephthalate and polypropylene, to be more common (Evangelos Danopoulos et al. 2020). Further, Danopoulos et al. (2020) highlight that the prevalent shapes of the MPs identified in water samples were fragments and fibers, rather than the pristine microspheres reported here.

Similar observations of the limitations in MPs toxicological literature have been made by other research teams, emphasizing the importance to address these issues in future undertakings (Coffin et al. 2022; Gouin et al. 2022). This work builds on previously reported recommendations by applying not only a survey of the quality criteria but also identifying potential RoB using a fit-for-purpose tool. Importantly, aspects of selection, performance, and attrition bias were integrated into the critical appraisal to better understand the potential bias encountered in these study domains.

Although the lack of these considerations is addressed by the validity tool developed and applied to this body of literature, no known validated protocol exists for MP (and specifically for reference materials). Creation of such a protocol or protocols is a recommendation for a path forward to strengthen the evidence. Use of validated test guidelines, such as those developed by OECD, would provide a more reliable body of evidence. To address these limitations, validated, gold-standard protocols should be developed for assessment of the potential effects of MP exposure on developmental and reproductive toxicity, with emphasis on the chemical identification and exposure parameters. The lack of this framework has been noted in a growing body of recent literature (Coffin et al. 2022; Gouin et al. 2022; Koelmans et al. 2022; WHO 2022; Duncan et al. 2024).

Although MPs research related to human health toxicity is growing rapidly, it is still in its infancy. Therefore, it is critical that researchers understand the basic toxicological principles of MPs prior to initiating further mandates based on low quality data; partnerships between polymer chemists, material/particle scientists, reproductive toxicologists, and analytical chemists on study design and conduct is highly recommended. The most critical features identified to improve reliability and reduce bias for reproductive toxicity studies on MPs are summarized below (Box 1).

Box 1. Critical features needed to improve confidence in reliability and reduce bias for reproductive toxicity studies on MPs.

- Utilize environmentally relevant test materials and document their justification.
- Fully characterize the test materials, including particle surface features and non-particle components.
- Confirm dose stability, suspension/homogeneity, and concentration in the test system covering the duration of the administration.
- Methods are sufficiently detailed so that study replication is possible.
- For any reproductive endpoint (functional, hormonal, structural), a detailed assessment of the general health of the parental unit is available for comparison.
- Assessment methods for effects endpoints are valid, reliable, and sufficiently robust to be consistent with the principles of the relevant regulatory test guidelines.

Beyond addressing these factors in future research, studies assessing the potential developmental and reproductive health effects of MP particles should either follow that which is recommended across various authoritative guidelines for the assessment of these outcomes or incorporate the specific guideline study designs and measures deemed relevant in these validated studies. These include a range of study protocols such as those developed by the USEPA (1996, 1998a, 1998b, 2000a, 2000b) and OECD (2001, 2007, 2016a, 2016b, 2018b). Collectively, addressing features in Box 1 and using harmonized and/or validated methodologies will allow for more confidence in assessing risk—including concepts such as the relevance of doses used in comparison to human exposures or the ability to develop health-based benchmarks using meta-analytical techniques.

Recent workshops held to discuss scientific advancements needed to support the evaluation of MPs effects testing and risk assessment have identified similar needs. For example, the Microplastics Reference Material workshop invited a range of experts to discuss the generation of micro- and nanoplastic particle reference materials (American Chemistry Council 2022). The participants identified several opportunities for the development of reference materials related to the size, shape, and homogeneity/heterogeneity of particles, along with approaches in how best to generate them. Although this discussion is ongoing and progress has been made in identifying the necessary parameters, the experts also concluded that developing the ideal reference material will likely take several years. Further, there is still a need to better characterize environmental exposures prior to the use of MP reference material in effects testing.

In October 2022 and July 2023, workshops were hosted by the International Council of Chemical Associations Microplastics Advance Research and Innovation Initiative, which aims to support communication and dialogue between all stakeholders engaged in MP research to enable advances in scientific understanding related to both the environmental and human health risks of MPs (https://icca-chem.org/focus/microplastics-advanced-research-and-innovation-initiative). Stakeholders included industry, government, and academic researchers who are developing and applying methods aimed at strengthening scientific understanding in MP risk assessment, with an emphasis on supporting science-based decision-making within a regulatory context. An important ongoing challenge identified from the discussions, and which continues to represent a barrier toward supporting science-based decision making in the context of risk assessment, is the lack of standard analytical methods and use of standard effect test systems. A key takeaway message from workshop discussions is that there is a broad consensus that the lack of reliable and relevant standard methods limits our ability to characterize and quantify both exposure and hazard.

The inconsistency between the types of MPs that are understood to characterize environmental exposure and those used in effect test systems is therefore representative of one of the most significant challenges in enabling a quantitative assessment of risk. To address this concern, there are several initiatives currently ongoing aimed at generating environmentally relevant reference MPs, including those supported by ACC, the Joint Research Centre, and the National Institute for Standards Testing, as well as within several EU H2020 projects and the Plastics Europe Brigid project. The generation of a suite of well-characterized reference MPs would provide opportunities to strengthen the development of analytical methods, whereas also helping to improve the quality and reliability of data generated from hazard testing. Ideally, access to an environmentally relevant suite of reference MPs would support risk characterization, with the properties of MPs most likely to represent a risk being systematically screened and prioritized. Some of the key recommendations from the workshop included a need for increased hazard testing using standardized methods with dosimetry as a component in the test systems, a need to develop and apply in vitro to in vivo extrapolation methods, toxicokinetics of MPs to evaluate and contextualize reported effects, support of research aimed at identifying appropriate negative and positive controls to include in effects test systems, QA/QC considerations, ensuring particle characteristics are evaluated and reported, and support of activities aimed at generating and making available relevant reference materials.

## Conclusion

This systematic review highlights significant limitations in the current body of evidence evaluating the reproductive and developmental toxicity of MPs in mammals. Critical methodological flaws leading to insufficient exposure characterization and critical gaps in outcome assessment result in a high RoB that undermines confidence in the findings for risk assessment and hinders the establishment of dose–response relationships. Additionally, discrepancies in test material characterization and exposure homogeneity raise concerns about reproducibility and comparability across studies. Moving forward, standardized methodologies, including validated reference materials, robust study designs, and adherence to internationally recognized testing frameworks/guidelines, are needed. Collaborative efforts among toxicologists, polymer scientists, and regulatory agencies will be essential to improve study quality and generate data suitable for human health risk assessment. Until these gaps are addressed, the current evidence base remains insufficient for deriving toxicological benchmarks or informing science-based regulatory decisions regarding MP exposure.

## Supplementary Material

kfaf108_Supplementary_Data
